# Bone Involvement in Hyperphosphatemic Familial Tumoral Calcinosis: A New Phenotypic Presentation

**DOI:** 10.5041/RMMJ.10445

**Published:** 2021-07-20

**Authors:** J. Daniel Freedman, Rostislav Novak, Sharon Bratman Morag, Emily Avitan-Hersh, David Nikomarov

**Affiliations:** 1 Department of Family Medicine, Henry Ford Health System, Detroit, Michigan, USA; 2 Orthopedic Surgery Section, Rambam Health Care Campus, Haifa, Israel; 3 Department of Internal Medicine H, Rambam Health Care Campus, Haifa, Israel; 4 Department of Dermatology, Rambam Health Care Campus, Haifa, Israel; 5 Musculoskeletal Oncology Surgery, Orthopedic Surgery Section, Rambam Health Care Campus, Haifa, Israel

**Keywords:** Calcinosis, mutation, pathologic fracture

## Abstract

Mutations in *FGF23*, *KL*, and *GALNT3* have been identified as the cause for the development of hyperphosphatemic familial tumoral calcinosis (HFTC). Patients with HFTC typically present in childhood or adolescence with periarticular soft tissue deposits that eventually progress to disrupt normal joint articulation. Mutations in the *GALNT3* gene were shown to account for the hyperphosphatemic state in both HFTC and hyperostosis-hyperphosphatemia syndrome (HHS), the latter characterized by bone involvement. We present the case of a patient of a Druze ethnic origin with known HFTC that presented to our department with the first documented case of pathologic fracture occurring secondary to the disease. Our report introduces this new phenotypic presentation, suggests a potential role for prophylactic bone screening, and highlights the need for preconception genetic screening in selected populations.

## INTRODUCTION

Hyperphosphatemic familial tumoral calcinosis (HFTC) is characterized by high levels of serum phosphate and subsequently by subcutaneous deposits of calcium-phosphate crystals. These non-cancerous, slowly growing, calcified masses accumulate mostly around larger joints, with a tendency towards the shoulders, elbows, and hips, although they can commonly involve the overlying skin.^1^ Additionally, these deposits have been reported to affect other soft tissues,^2^–^4^ including muscular compartments, fascial layers, and adipose tissue. These masses may initially be asymptomatic, but as they become larger, they can interfere with joint articulation. Onset typically occurs in childhood or adolescence,^1^ and is common in people of Middle Eastern and African descent.^5^ The condition is genetically inherited via an autosomal recessive pattern.^6^ There are two distinct forms of the disease: HFTC and normophosphatemic familial tumoral calcinosis (NFTC), which are differentiated by serum phosphate levels. Blood calcium levels are usually within the normal range in both cases. The defect in NFTC is related to SAMD9, a tumor suppressor and anti-inflammatory protein.^1^ Normophosphatemic familial tumoral calcinosis (NFTC) is less prevalent than HFTC and typically presents in the first year of life as a non-specific erythematous rash and mucosal inflammation.^1^ Hyperphosphatemic familial tumoral calcinosis (HFTC) has been demonstrated to result from a genetic mutation in one of the three phosphate-regulatory genes *FGF23*, *KL*, and *GALNT3* (MIM #617993, #617994, #211900), though most commonly identified in the last-mentioned. The *FGF23* gene encodes for fibroblast growth factor-23, which is a phosphaturic protein and is produced in bone cells. The other two genes *KL* and *GALNT3* help to regulate FGF23. The *KL* gene encodes the Klotho protein and serves as co-receptor for FGF23 which prevents phosphate reabsorption in the proximal renal tubule. The *GALNT3* gene encodes a glycosyltransferase protein, ppGalNacT3, and is responsible for O-glycosylation of FGF23, allowing FGF23 to migrate from the cell, thereby preventing its breakdown.^1^ A mutation in any of these proteins will disrupt FGF23 signaling, causing impaired phosphate excretion. Calcinosis results when the increased serum phosphate levels interact with calcium. Therapy includes both supportive and surgical treatments, though there is no definitive cure. Debulking may provide symptomatic relief but often involves multiple subsequent operations due to incomplete removal and high risk of recurrence.^7^ The purpose of this case report is to review this rare genetic condition and to describe its possible role in causing pathologic fractures.

## CASE PRESENTATION

**Figure 1:**
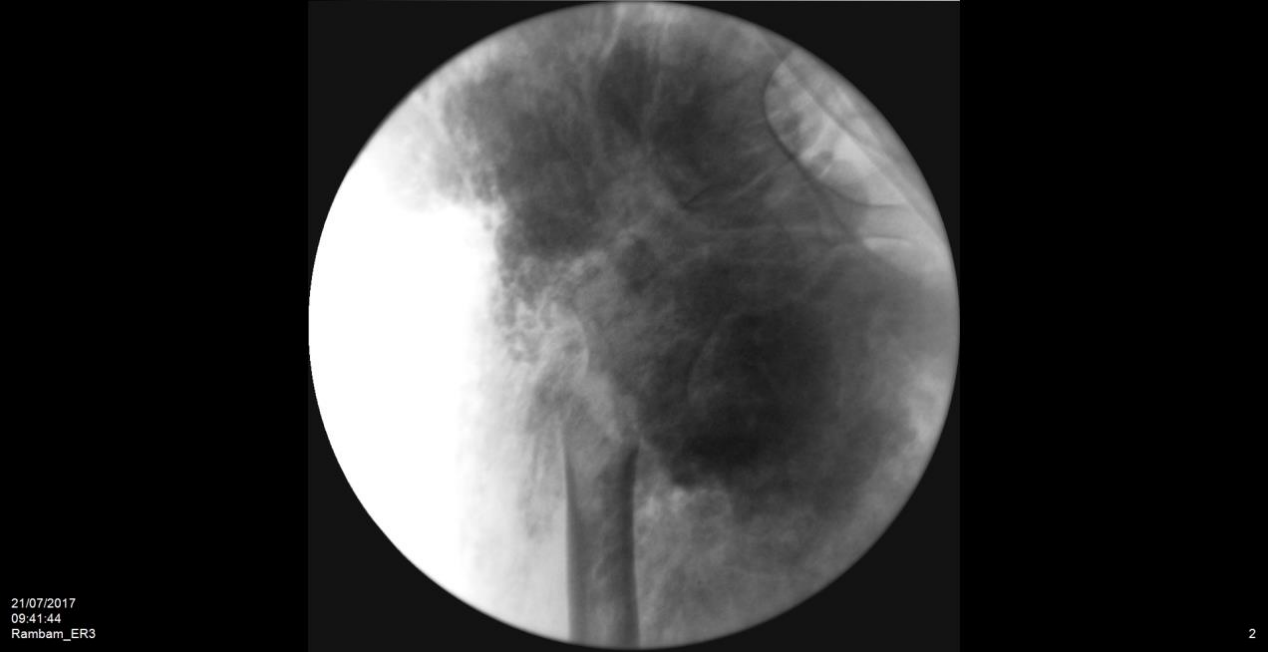
Periarticular Soft-tissue Deposits and Pathological Subtrochanteric Fracture.

**Figure 2:**
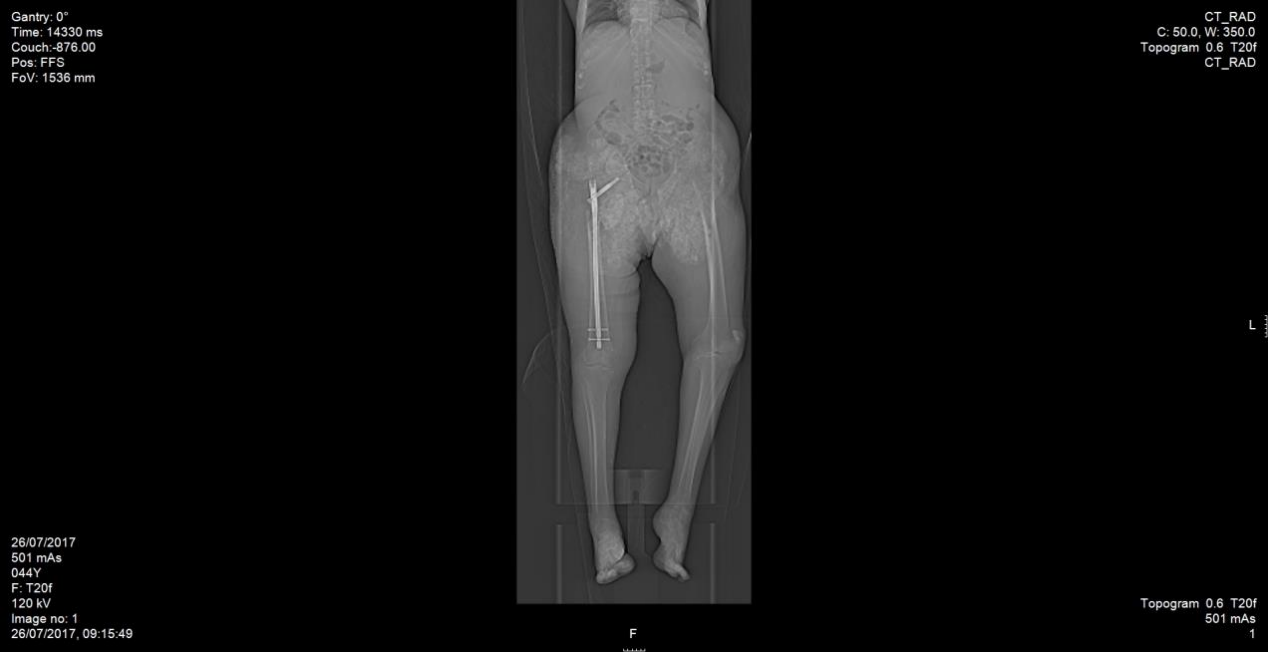
Post-operative CT Scan. During the operation, periarticular calcium deposits made the visualization under X-ray fluoroscopy impossible. Therefore, debulking, open reduction, and internal fixation were mandatory.

We present the case of a 42-year-old male of a Druze ethnic origin, with a known diagnosis of HFTC as a result of a familial mutation in *GALNT3*. The patient was clinically diagnosed during his childhood with HFTC, and a molecular analysis revealed a splice site mutation that resulted in the disruption of the intron 8/11 donor site in *GALNT3*. Previous studies have described our patient’s familial mutation, though at the time no bone pathology was observed.^6^,^8^ The patient presented for the first time in adolescence with periarticular involvement beginning around the hips, which later progressed to involve the shoulders, elbows, and jaw. He went through over 20 marginal resections of calcified masses in different parts of his body. In 2017, the patient was brought to our department with a deformed right leg. Following X-ray imaging, subtrochanteric fracture was diagnosed (Figure 1). The patient denied any significant trauma prior to presenting to our department, but endorsed two months of persistent right hip pain before diagnosis. Prior to the operation, the patient underwent systemic evaluation to rule out any oncologic reason for the pathological fracture. The fracture was subsequently surgically repaired with open reduction and internal fixation (Figure 2). During the operation, specimens were collected and sent to pathology, which subsequently ruled out primary and secondary (metastatic) bone tumors. Upon further worsening of the underlying disease, the calcinosis invaded to his larynx, causing respiratory compromise and resulting in tracheostomy. The patient died two months later following a systemic infection and multiple organ failure.

## DISCUSSION

We believe this is the first report of a pathologic fracture secondary to HFTC in the literature. While periarticular bony involvement is a common feature of HFTC, direct involvement of the bone is rare. Bone involvement is, however, common in hyperostosis-hyperphosphatemia syndrome (HHS). Hyperostosis-hyperphosphatemia syndrome is characterized by hyperostosis and recurrent bone lesions, which occur as a result of hyperphosphatemia. The characteristic radiographic features of affected bones include periosteal reaction, cortical hyperostosis, and diaphysitis.^6^,^9^ Clinical presentation of HHS was discovered to originate from a mutation in *GALNT3*, the same gene as HFTC, and, as such, the two diseases were considered allelic disorders.^6^ Occurrences of HFTC and HHS have even been reported in the same family.^3^,^10^ Therefore, HHS is now thought to be a mild variant of HFTC.^9^,^11^ While HFTC and HHS exert their effects primarily in two different tissues, namely soft tissue and bone, respectively, they were also found to co-manifest in the same patient.^2^,^11^ A recent literature review demonstrated more subjects with a combined HFTC-HHS phenotype than initially thought.^3^ Furthermore, a recent study reported a patient that presented with signs of HHS but later progressed to a predominantly HFTC phenotypic presentation, thereby concluding that the two conditions represent a spectrum of the same disease.^9^ Although our patient was initially diagnosed with HFTC due to clinical manifestations, hyperphosphatemia, and a mutation in *GALNT3*, it is conceivable that the described bone involvement in the setting of pathologic fracture places him on the HFTC-HHS spectrum, thereby contributing another patient with this rare combined phenotype to the small patient pool.

The GALNT3 (c.1524+1G>A) variant was discovered in families of Druze ethnic descent^8^ and was later identified in Arab Muslim families with HHS.^6^ This sequence is highly conserved in all species, thus highlighting the importance of the mutation. A haplotype analysis of this population estimated an allele frequency of 0.70%, and a likely founder effect with the mutation arising approximately 88 to 200 years ago.^6^ This also suggests a genetic founder linkage between Muslim and Druze communities in the Middle East and a tight genetic correlation between HFTC and HHS. These new findings amplify the need to screen patients’ families for the genetic mutation as well as possible bony involvement in order to avoid future fracture complications.

Our patient underwent numerous debulking procedures due to symptomatic tumoral calcinosis. He was not treated medically, as no definitive treatment currently exists, and only case reports and case series have been published on this rare condition. Certain reported treatments have attempted to lower serum phosphate levels, though with inconsistent results. These include a low-phosphate diet, phosphate chelation, as well as medications promoting the renal excretion of phosphate.^7^ Recently, a prospective study has demonstrated a promising role for sodium thiosulfate, a drug primarily used to treat cyanide poisoning, as a treatment for ectopic calcification resulting from HFTC and HHS.^12^ Due to the recurrence potential of the disease, this treatment option should be considered for symptomatic relief.

The importance of identifying a fracture occurring secondary to HFTC-HHS demonstrates the need to monitor patients with bone involvement and severe periarticular calcinosis for impending fracture. Repair of the fracture was especially difficult in our case with regard to preoperative planning and intraoperative navigation due to limited X-ray penetration through periarticular calcifications. Thus, the operation was performed as an open reduction and internal fixation. The fracture site was opened in order to visualize and reduce the fracture appropriately. Due to the complexity of the operation that occurred later in the course of disease, we suggest actively monitoring patients with periarticular bone involvement to rule out impending fracture. Should additional similar cases be reported, prophylactic surgical correction with intramedullary fixation and bone stabilization may help to prevent such late, complicated fracture repair.

## CONCLUSION

We have described a case report of a Middle Eastern male patient of Druze ethnic descent, with a *GALNT3* mutation, presenting with the first documented case of pathologic fracture secondary to this mutation. This presentation highlights the importance of monitoring bone involvement in HFTC patients with *GALNT3* variants due to the potential co-occurrence of HHS, and demonstrates the need to identify impending pathologic fractures in order to prevent future complications. Due to high allele frequency and a possible founder effect, this variant should be considered for preconception genetic screening, especially in segregated societies.

## Abbreviations

HFTC: hyperphosphatemic familial tumoral calcinosis
HHS: hyperostosis-hyperphosphatemia syndrome
